# Borophene Embedded Cellulose Paper for Enhanced Photothermal Water Evaporation and Prompt Bacterial Killing

**DOI:** 10.1002/advs.202205809

**Published:** 2023-01-25

**Authors:** Xinwei Guan, Prashant Kumar, Zhixuan Li, Thi Kim Anh Tran, Sumit Chahal, Zhihao Lei, Chien‐Yu Huang, Chun‐Ho Lin, Jing‐Kai Huang, Long Hu, Yuan‐Chih Chang, Li Wang, Jolitta S. J. Britto, Logeshwaran Panneerselvan, Dewei Chu, Tom Wu, Ajay Karakoti, Jiabao Yi, Ajayan Vinu

**Affiliations:** ^1^ Global Innovative Centre for Advanced Nanomaterials (GICAN) College of Engineering, Science and Environment (CESE) The University of Newcastle Callaghan NSW 2308 Australia; ^2^ Department of Physics Indian Institute of Technology Patna Bihta Campus Patna 801106 India; ^3^ School of Materials Science and Engineering University of New South Wales (UNSW) Sydney NSW 2052 Australia; ^4^ School of Photovoltaic and Renewable Engineering the University of New South Wales Sydney NSW 2052 Australia; ^5^ Department of Applied Physics The Hong Kong Polytechnic University Hung Hom Hong Kong 999077 China

**Keywords:** antibacterial, borophene, cellulose nanofibers, photothermal, water evaporation

## Abstract

Solar‐driven photothermal water evaporation is considered an elegant and sustainable technology for freshwater production. The existing systems, however, often suffer from poor stability and biofouling issues, which severely hamper their prospects in practical applications. Conventionally, photothermal materials are deposited on the membrane supports via vacuum‐assisted filtration or dip‐coating methods. Nevertheless, the weak inherent material‐membrane interactions frequently lead to poor durability, and the photothermal material layer can be easily peeled off from the hosting substrates or partially dissolved when immersed in water. In the present article, the discovery of the incorporation of borophene into cellulose nanofibers (CNF), enabling excellent environmental stability with a high light‐to‐heat conversion efficiency of 91.5% and water evaporation rate of 1.45 kg m^−2^ h^−1^ under simulated sunlight is reported. It is also demonstrated that borophene papers can be employed as an excellent active photothermal material for eliminating almost 100% of both gram‐positive and gram‐negative bacteria within 20 min under three sun irradiations. The result opens a new direction for the design of borophene‐based papers with unique photothermal properties which can be used for the effective treatment of a wide range of wastewaters.

## Graphical abstract

A novel borophene‐embedded cellulose paper is fabricated by incorporating two‐dimensional borophene nanosheets inside the cellulose fiber structure. The borophene paper exhibits outstanding absorption properties, featuring excellent stability and mechanical properties for photothermal water evaporation and bacterial killing applications.

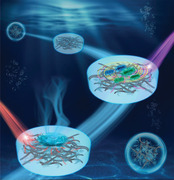

## 1 Introduction

In modern society, fresh water is still precious and a scarce resource in many parts of the world. The consumption of fresh water has significantly increased recently due to the combination of several issues, including explosive expansion of the human population, rapid industrial development, and severe environmental pollution.^[^ ^1^ ^]^ It was recorded that only Sydney alone used more than 600 gigalitres of water in 2018. In this context, solar‐thermal technologies, which utilize solar energy and photothermal materials to generate steam, have drawn significant attention as a renewable and environment‐friendly approach for clean‐water production, thanks to their low‐cost, high adaptability, excellent portability, and low energy consumption.^[^ ^2^, ^3^, ^4^, ^5^ ^]^

Among all the solar‐driven water evaporation designs, localized heating on the water liquid‐vapor interface is regarded as one of the most simple yet efficient strategies for water evaporation, delivering fast temperature rise, high evaporation rate, and full exploitation and absorption of the solar spectrum.^[^ ^6^, ^7^ ^]^ In the last decade, a large number of novel photo‐absorbing materials have been explored and investigated as photothermal agents, such as carbon‐based nanomaterials,^[^ ^8^, ^9^ ^]^ plasmonic metal nanoparticles,^[^ ^10^, ^11^ ^]^ two‐dimensional (2D) materials,^[^ ^12^, ^13^, ^14^ ^]^ and black metal oxides,^[^ ^15^, ^16^ ^]^ in which these materials are generally deposited on the surface of floating membrane via vacuum‐assisted filtration or dip‐coating methods. However, a significant drawback of this device architecture lies in the weak inherent interactions between the support membrane and photothermal materials, especially when immersed in non‐static water.^[^ ^13^ ^]^ As a result, the deposited materials could be dissolved in water or peeled off from the hosting substrate easily, resulting in poor durability and stability of the photothermal system.^[^ ^12^, ^13^ ^]^ Moreover, the high density of photothermal materials on the surface of membranes is prone to block the water channel and further reduce the water supply, leading to the destruction of the membrane and a decrease in the evaporation rate.^[^ ^17^ ^]^ Another potential issue of long‐term stable photothermal water evaporation is the biofouling conundrum, which requires the implementation of toxic chemicals for bacterial killing and severely shortens the photothermal membrane lifetime.^[^ ^18^ ^]^ It has been well acknowledged that the existence of pathogenic bacteria in water purification systems badly threatens human health.^[^ ^19^ ^]^ Therefore, the development of novel photothermal materials with unique structures and designs is imperative to expand their lifetime and enhance antibacterial performance for practical solar‐driven water evaporation.

Recently, our previous work conceived a novel approach to vacuum filtration growth of boron nitride (h‐BN) with cellulose fiber (CNF) at room temperature.^[^ ^20^ ^]^ Rather than depositing 2D h‐BN on the surface of cellulose paper, h‐BN nanosheets were homogenously incorporated inside the fibrillated structure, empowering a high thermally conductive h‐BN paper with extraordinarily high stability and flexibility. We believe such a technique is also suitable for photothermal membrane synthesis since the cellulose fibers can not only act as the supporting substrate but also protect the agent materials. It should be mentioned that such bio‐based fibrous materials have recently emerged as superstars for highly efficient electrochemical applications from energy to environment and health monitoring, which might replace conventional non‐renewable materials in future technologies.^[^ ^21^ ^]^ Meanwhile, our group successfully exfoliated freestanding borophene (the lightest 2D material) via sonochemical and micromechanical methods, which considerably extend their applications,^[^ ^22^, ^23^ ^]^ including Li‐/Na‐ion batteries,^[^ ^24^, ^25^, ^26^ ^]^ electrocatalysis,^[^ ^27^ ^]^ and sensors.^[^ ^22^, ^28^, ^29^ ^]^ As the lightest elemental Dirac 2D boron allotrope, borophene has various advantages, such as high predicted high mobility and thermal conductivity, broadband absorption from entire UV to FIR, and ultra‐fast luminescence, enabling borophene an ideal material for solar‐driven photothermal applications.^[^ ^30^ ^]^ Borophene is metallic for its two prominent phases *β*_12_ and X_3_. It is however semiconducting in its *α* phase. At the core of the extraordinary behavior of borophene is the lack of a sufficient number of electrons in boron atoms to have covalent bonding. Boron exhibits unusual bonding nature (3‐center‐2‐electron bond). 3D‐protrusion which is common to X_3_ and *β*_12_ phases of borophene arises in order to stabilize energetically. Its *β*_12_ phase in particular has an anisotropic crystal structure, which gives anisotropic electronic as well as thermal conductivity and elastic moduli. Correlation between out‐of‐plane and in‐plane vibrations in borophene and the role of phonon‐photon coupling in a very special crystallographic structure of borophene to exploit its photothermal effect has never been explored and therefore is very promising from a novelty point of view and for its potential practical applications. The present manuscript explores the photothermal water evaporation and bacterial killing application of borophene embedded in flexible cellulose paper, which thus opens up the application of borophene for purifying water from various microbes in an effortless manner.

**Scheme 1:**
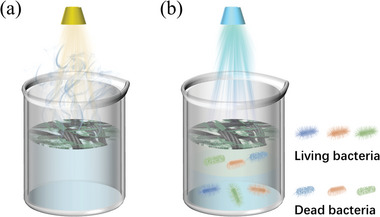
Schematic illustrations of the a) photothermal water evaporation and b) photothermal bacterial killing, based on borophene‐embedded cellulose papers.

In this work, we adopt this facile, room‐temperature, solution‐processed method to synthesize highly durable and flexible borophene/CNF hybrid paper for the first time, elucidating their key physical properties and further demonstrating their potential in photothermal water evaporation and killing of bacteria (**Scheme** **1**). Compared to the traditional photothermal systems in which materials are deposited on the membrane supports with weak inherent interaction, the borophene is intimately inserted inside the hosting CNF structure. The hybrid paper demonstrates a high water evaporation rate of 1.45 kg m^−2^ h^−1^ and light‐to‐heat conversion efficiency of 91.5%, with superior mechanical stability inside water and maintaining more than 90% of the initial water evaporation rate for one month. Notably, the borophene papers also demonstrate extraordinary antibacterial performance, eliminating almost 100% of bacteria within 20 min under three sun irradiation. The fabrication process does not require any advanced technology, and the fabricated borophene papers can be brought anywhere in the world and used to get clean water and kill materials under light illumination.

## 2 Results

**Figure 1:**
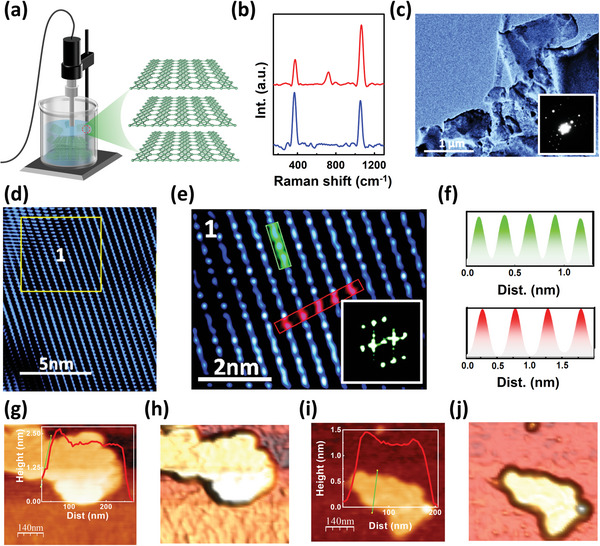
a) Schematic illustration of sonochemical exfoliation for preparing 2D borophene. b) Raman spectrum of borophene. The red curve represents bilayer borophene with identification peaks at 375, 725, and 1068 cm^−1^. The 725 cm^−1^ peak disappears for monolayer sheets (blue curve). c) TEM images of exfoliated borophene nanosheets. d, e) HRTEM images and the zoomed‐in image with clearly visible outward and inward atoms. f) The average interatomic distances at two locations marked in (e) were 0.25 and 0.50 nm (corresponding to the marked red line). g, i) AFM image of borophene nanosheet with layer heights of 2.1 nm (5 layers) and 1.3 nm (3 layers), respectively. The insets show corresponding line profiles, and their 3D views are shown in h) and j).

Freestanding borophene was synthesized by liquid‐phase sonochemical exfoliation according to previously reported procedures.^[^ ^22^ ^]^ In brief, highly crystalline boron powder was sonochemically exfoliated by a probe ultrasonicator in N,N‐Dimethylmethanamide (DMF) solvent for 12 h (**Figure** **1**a). The high power of our probe ultrasound over 1 kW ensures a thorough conversion from bulk boron into mono‐/multilayer borophene nanosheets with an ordered crystalline structure. Raman characterizations of borophene nanosheets reveal the existence of the mixed phase. Essentially, we attained A^2^_g_ (∼1068 cm^‐1^) and A^3^_g_ (∼725 cm^‐1^) Raman modes of *β*_12_ phase and B^2^_1g_ peak (∼375 cm^‐1^) of X_3_ phase in sample attained at 3000 RPM centrifugation speed which exhibited 4–6 layers of borophene (Figure 1b). When centrifugation speed was enhanced to 4000 RPM, samples exhibited 1–3 layers, and we could see a clear difference (middle A^3^_g_ peaks disappeared) in the Raman spectrum. Transmission electron microscopy (TEM) images further confirm the existence of borophene nanosheets with 3–5 µm lateral dimensions (Figure 1c). The large‐area high‐resolution TEM (HRTEM) image (Figure 1d) and the zoomed‐in image of marked regions (Figure 1e) further confirm the excellent crystalline quality of borophene. The top‐lying ridgelines of atoms (visible in Figure 1e) and bottom‐lying atoms (not visible in Figure 1e) between the two ridgelines correspond to the *β*_12_ phase of borophene.^[^ ^29^ ^]^ In the liquid phase exfoliation of boron crystal, various phases of borophene form, including prominent phases such as stripe, *β*_12,_ and X_3_ phases. The atomic line profile exhibits an inter‐atomic distance of 2.5 Å along the ridgelines and 5 Å normal to it (Figure 1f). The layered nature and corresponding thicknesses of the borophene nanosheets are verified by atomic force microscopy (AFM) characterizations, as demonstrated in Figure 1g‐j. Borophene nanosheets have lateral dimensions from a few hundred nanometers to larger than 1 µm with varied thicknesses from 0.4 nm (monolayer) to 1.6 nm (four layers) as observed in line profiles (inset images in Figure 1g,i), which can be isolated via centrifugation under different conditions. A higher RPM condition (>3000 RPM) leaves only monolayers or a few monolayers in the supernatant. The supernatant is isolated for further usage.

**Figure 2:**
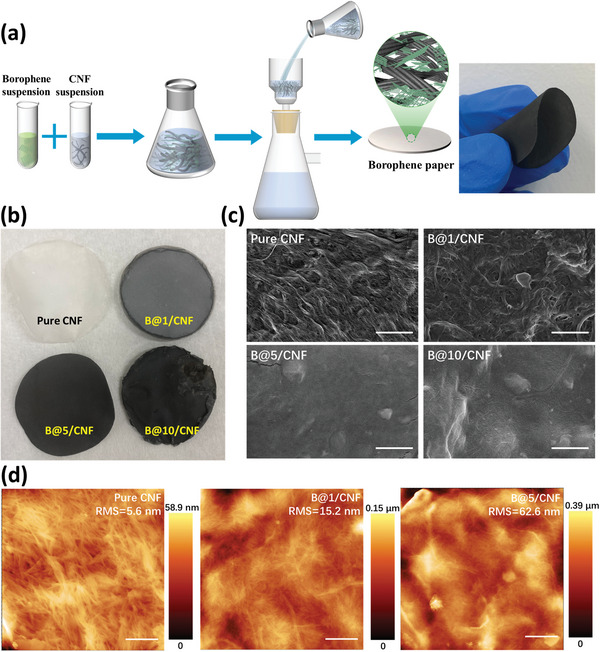
a) Schematic diagram of the fabrication process of the borophene/CNF paper. The right photography demonstrates its high flexibility. b) Digital photos of borophene/CNF papers with different borophene concentration from 0 mg, 1 mg, 5 mg, and 10 mg. Top‐view c) SEM and d) AFM images of borophene/CNF papers with different concentrations. The scale bars are 1 µm.

The detailed synthesis procedures of the borophene paper are illustrated in **Figure** **2**a. In the beginning, commercial bleached softwood pulp was treated by an oxidation process, followed by dissolving in DMF solvent to generate CNF suspension.^[^ ^20^ ^]^ Subsequently, the raw CNF‐suspended solution was further disintegrated by a microfluidizer to obtain the fine CNF suspension composed of 1D fibrils.^[^ ^31^ ^]^ Afterward, the previously prepared 2D borophene dissolved in DMF was mixed with the CNF suspensions in a sonic bath, and then the solution was vacuum‐filtered and dried through a 20 nm ceramic filter for 12 h to entirely remove the DMF solvent and thus finally obtaining borophene papers. The solid‐state paper is robust and flexible, just like daily used papers. In order to optimize the optical and photothermal properties of borophene paper, varied amounts of 20‐µm boron powder (i.e., 0, 1 mg, 5 mg, and 10 mg, labeled as pure CNF, B@1/CNF, B@5/CNF, and B@10/CNF, respectively) were firstly exfoliated via sonochemical approach using an ultrasonic probe in DMF solvent for 12 h. A digital photo and structure diagram of highly‐flexible borophene papers are presented in Figure 2b, demonstrating the color evolution of borophene papers with different concentrations. In this structure, 2D borophene nanosheets stack randomly along the in‐plane direction of the paper. The large contact area between the borophene nanosheets assists in heat transport, and appropriate amounts of borophene will not block the water channel through the capillary effect, ensuring a stable water supply. In contrast, when we deposited borophene only on the surface of cellulose paper, we observed that borophene powder could be easily wiped out by scratching (Figure S1, Supporting Information), demonstrating poor integrity and durability of this device structure.

Notably, mixing overloading amounts of borophene with CNF could counteract the advantage of this method because extra borophene would float on the surface of the paper without the protection of CNF. During our vacuum‐filtering process, we found that 10 mg of borophene led to a filtering difficulty, which took more than a few hours to entirely remove DMF solvent from the solution, compared to only several minutes when borophene weight is <5 mg. In addition, from Figure 2b, we observe that the surface of B@10/CNF paper is much rougher than the other three papers, and the color is not homogeneous even though it is slightly darker, possibly due to the precipitation of borophene during the extended time filtration process. The top‐view scanning electron microscopy (SEM) images in Figure 2c clearly illustrate this trend. Pure CNF presents an entangled 1D fiber structure; with increasing the borophene concentration, the fiber‐connected surface is gradually covered by a layer of borophene nanosheets, and a rougher morphology can be observed in the B@10/CNF paper. AFM characterizations in Figure 2d and Figure S2 (Supporting Information) further confirm the hypothesis that these hybrid papers gradually become rougher as the borophene concentration increases, with the root mean square (RMS) roughness of 5.6, 15.2, 62.6, and 128.5 nm for bare CNF, B@1/CNF, B@5/CNC, and B@10/CNC, respectively, indicating that higher concentration of borophene significantly alters the morphology of hybrid papers. Although a rougher surface may microscopically generate more reflections inside the material and improve light absorption, the mechanical durability and stability of the paper can be severely affected. Since a higher concentration of borophene leads to extra sedimentation on the surface of CNF paper, a certain amount of borophene can float on the surface of the hybrid paper without the interaction of CNF. Therefore, the stability of B@10/CNF is expected to be lower compared to B@5/CNF and B@1/CNF. From AFM and SEM images, it is clear that 2D borophene nanosheets are compactly wired by the 1D CNF, providing strong mechanical strength and reliable durability for photothermal applications. In addition, the ultrathin 2D nature of borophene nanosheets can retain the flexibility of the borophene paper, as demonstrated by the TEM in Figure S3a (Supporting Information). The borophene is well incorporated and homogeneously distributed inside CNF, and the electron diffraction pattern of B@5/CNF is shown in the inset of Figure S3b,c, Supporting Information, verifying the high crystallinity of borophene embedded in the CNF network.

As borophene is not simply lying on the paper's surface but is tightly incorporated inside the CNF structure, excellent structural integrity can be ensured with appropriate borophene concentrations. Figure S4 (Supporting Information) exhibits excellent structural integrity of B@5/CNF paper after either 1 h of ultrasonication in the lab as well as one month of water immersion outdoors. No visible black material or paper scraps can be observed in water, suggesting superior mechanical integrity and durability of the borophene paper thanks to strong material‐CNF interactions.^[^ ^32^ ^]^ However, for the B@10/CNF sample, we can clearly see black floating materials immediately just by gently shaking the solution (Figure S4, Supporting Information). In addition, we tested the structural integrity of pure CNF, B@1/CNF, B@5/CNF, and B@10/CNF papers when immersing in water, as shown in Figure S5 (Supporting Information). It is clear that all pure CNF, B@1/CNF, and B@5/CNF papers can retain structural integrity in water for ten days, suggesting our borophene papers have excellent stability for practical applications. Nevertheless, the B@10/CNF paper began to decompose after two days, indicating that an overloaded concentration of borophene can detrimentally affect the paper's structural integrity.

**Figure 3:**
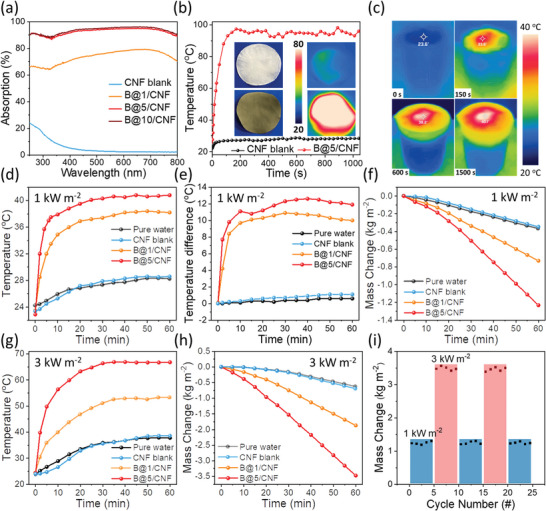
a) UV‐Vis absorption spectra of blank CNF and borophene papers with different concentrations. b) Temperature time course of pure CNF paper and B@5/CNF paper in the air under one sun irradiation. Inserts are their IR photos after irradiating for 200 s. c) IR images of B@5/CNF paper floating on water surface under one sun irradiation for varied times. d) Temperature variations of the surface of pure water, blank CNF paper, B@1/CNF paper, and B@5/CNF paper under one sun solar irradiation, and the corresponding temperature difference between the top paper surface and beaker's bottom of each sample are shown in (e). f) Evaporation water mass changes against time of these samples under one sun irradiation. g) Temperature variations of samples under three sun irradiation. h) Water mass changes against time of these samples under three sun irradiation. i) Evaporation cycle tests of the B@5/CNF paper under one and three sun illumination. Each cycle lasts for one hour.

UV‐Vis absorption of photothermal materials is a key factor for the potential photothermal performance with high solar‐to‐heat conversion.^[^ ^6^ ^]^ Incorporating 2D borophene into CNF paper not only delivers good mechanical flexibility and stability, but also significantly improves light absorption. As shown in **Figure** **3**a, pure CNF paper exhibits high transparency with less than 10% absorption in the visible regime, while the B@5/CNF and B@10/CNF papers absorb more than 90% of light across the visible regime, ensuring the full exploitation of solar energy. The B@1/CNF paper exhibits a relatively lower light absorption of ≈ 70%, which is ascribed to insufficient covering with a marginal amount of borophene loading. An infrared (IR) camera (FLIRONE PRO) was utilized to monitor the temperature change and thermal distribution of papers in the air and floating on the water under illumination. Figure 3b illustrates the temperature rise of the B@5/CNF paper and the bare CNF paper in ambient conditions under one sun illumination. As expected, the temperature of B@5/CNF paper rapidly jumped upward within three minutes and reached a high equilibrium temperature of ≈ 95 °C, while the bare CNF paper only achieved ≈ 29 °C at equilibrium after 1000 s. Such a rapid and high‐temperature increase of borophene paper can be attributed to borophene's low thermal conductivity. According to the value of electromagnetic power density *Q*, i.e., $Q=\frac{1}{2}\sigma_{r}{\left|E\right|}^{2}$ in the transient thermal evolution equation,^[^ ^33^ ^]^ we get:

(1) $$\rho c\;\frac{\partial T\left(r,t\right)}{\partial t}=\nabla \cdot \left(\kappa \nabla T\left(r,t\right)\right)+\frac{1}{2}\sigma_{r}{\left|E\right|}^{2}$$

where *ρ* is mass density, *c* is specific heat capacity, *κ* represents the thermal conductivity, *T* is the time (t) and space (r) dependent temperature, *σ*_r_ is the real part of surface electrical conductivity, and E is the electric field component of solar light. In addition, from the convection boundary condition, we can get:

(2) $$-\kappa \nabla T=h\left(T-T_{0}\right)$$

in which h is the convective heat transfer coefficient. Since convection terms will remain the same when the surface is changed, therefore, surface conductivity and thermal conductivity will essentially determine the photothermal evolution rate $\frac{\partial T(r,t)}{\partial t}$. The theoretical lattice thermal conductivity of borophene is 90 W mK^−1^ and 512 W mK^−1^ for *β*_12_ and X_3_ phases, respectively^[^ ^34^ ^]^; in contrast, graphene has a very high thermal conductivity of ≈ 5000 W mK^−1^.^[^ ^35^ ^]^ However, for pure samples, borophene and graphene have comparable electrical conductivity, yet borophene has an order of magnitude smaller thermal conductivity, which is why borophene is expected to have an enhanced photothermal effect, rendering it promising for solar‐driven interfacial water evaporation and photothermal disinfection applications.

The temperature evolution of the B@5/CNF paper floating on the water under one sun illumination is depicted in Figure 3c. The initial environment temperature is in the range of 23–24 °C, and the temperature of B@5/CNF at the water‐air interface rapidly increased to 33.6 ^o^C, 38.2 °C, and 40.1 °C after 150 s, 600 s, and 1500 s, respectively. Intriguingly, a strong localized heating effect is observed from side‐view IR thermal images of beakers with a temperature difference between top and bottom areas of more than 10 °C after 3 min of light illumination (Figure S6, Supporting Information), which considerably boosts steam generation by preventing heat loss due to bulk water heating. In contrast, the temperature difference was negligible in a control experiment with pure CNF paper, hinting that the localized heat should be arising from the harvest of solar energy by borophene paper rather than the blocking of direct sun irradiation by CNF (Figure S6, Supporting Information). Apparently, a more significant and fast temperature increase can be expected with the increase of borophene concentration due to solar light absorption enhancement. As shown in Figure 3d, the temperature changes of pure water, CNF, B@1/CNF, and B@5/CNF papers were recorded under one sun irradiation, achieving equilibrium temperatures of ≈ 28 °C, 28 °C, 38 °C, and 41 °C, respectively. The corresponding temperature difference (Figure 3e) between the top (air‐water interface) and bottom of the beaker also rapidly increased and reached 1 °C, 1 °C, 10 °C, and 12 °C, respectively, after 20 min of irradiation. Consequently, the corresponding mass reduction due to water evaporation is gradually enhanced from CNF paper to B@5/CNF, and water evaporation at 60 min could reach 1.3 kg m^–2^ for the B@5/CNF paper. Remarkably, the B@5/CNF exhibits excellent performance stability inside water, which retains the absorption properties and more than 90.9% of the initial water evaporation abilities after one month (Figure S7, Supporting Information).

It should be mentioned that even though the B@10/CNF could achieve a higher equilibrium temperature of ≈ 44 °C under the same experimental conditions, its water evaporation mass change value was not further improved but even slightly reduced compared to the B@5/CNF sample (Figure S8, Supporting Information), presumably because of the blocking of water transport channels by excessive loading of borophene (Figure 2c). During the synthesis, excessive borophene nanosheets caused the difficulty of vacuum‐filtering for the B@10/CNF hybrid paper, and its stability and homogeneity are also inferior as compared to the B@5/CNF, as aforementioned. Therefore, we abnegate the B@10/CNF paper for the following investigation.

The photothermal abilities of borophene papers were also examined under three sun irradiations. Remarkably, the surface temperature of the B@5/CNF paper rapidly approached 67 °C within half an hour (Figure 3g), and the top‐bottom temperature difference elevated to more than 37 °C accordingly (Figure S9, Supporting Information). We could observe a clear steam column under this set of experimental conditions, revealing the efficiency of our borophene papers for potential photothermal water evaporation applications. It should be mentioned that the temperature difference subsequently narrowed to 34 °C because of the massive thermal transfer resulting in a slow temperature rise of bulk water. In this experimental condition of three sun irradiations, the water evaporation rate of borophene papers is considerably higher than that under one sun condition, delivering 3.5 kg m^−2^ at 60 minutes for the B@5/CNF paper. The evaporation rates of different samples under one sun and three sun irradiations range from 1.45 to 3.88 kg m^−2^ h ^−1^, as summarized in Table S1 (Supporting Information). The high evaporation rate should be attributed to the uniform distribution of borophene on the CNF framework, which can facilitate the heat exchange between borophene and water. In addition, CNF can act as the channel for water evaporation, resulting in a high evaporation rate. Furthermore, we calculated the water evaporation efficiency (*η*) by the equation: $\eta =\frac{\frac{\mathrm{dm}}{\mathrm{dt}\times S}\times H_{e}}{Q_{S}}\times 100\%$, where m is the mass of evaporated water, t is the time, S is the surface area of the paper (1.5 × 10^−3^ m^2^), H_e_ is the heat of evaporation of water (≈ 2260 kJ kg^−1^), *Q*_s_ is the power density of the light source (1 Sun: 1 kW m^−2^; 3 Sun: 3 kW m^−2^). The B@5/CNF sample exhibits *η* values of 91.5% and 81.2% under one sun and three sun irradiation conditions, respectively, suggesting that the photothermal performance of our designed borophene paper is competitive and promising in comparison with other conventional and 2D photothermal (**Table** **1**), which can be attributed to its broadband absorption from entire UV to FIR, good photothermal conversion efficient, large specific surface area, and suitable thermal conductivity.

**Table 1:** Comparison of photothermal performance of the borophene paper and other prevailing materials

Photothermal system	Power density (kW m^−2^)	Efficiency (%)	Evaporation rate (kg m ^−2^ h ^−1^)	Year	Ref.
Al NP/AAM structure	4	88.4	5.7	2016	[36]
SWNT‐MoS_2_	5	91.5	6.6	2017	[37]
rGO/MCE	1	75	0.838	2017	[38]
Ti_2_O_3_ NPs	1	92.1	1.32	2017	[15]
MXene/PVDF	1	84	1.17	2017	[13]
PMoS_2_‐CC	1	80.5 ± 3.5	1.3	2018	[39]
HN/CNT photothermal paper	1	83.2	1.09	2018	[40]
BiInSe_3_@CF device	1	NA.	1.1	2018	[14]
MXene/filter membrane	1	71	1.31	2018	[41]
TiO_2_ porous layer	1.5	59.42	1.42	2019	[42]
Co_2.67_S_4_ NPs/PTFE	2	82	2.62	2019	[17]
MXene/cellulose fibrous	1	85.8	1.44	2019	[18]
1D‐OMoSNSA	1	89.6	2.5	2020	[43]
Ultrablack Silicon Structures	1	72.96	1.16	2021	[44]
Acetylene black membrane	1	90.46	1.58	2021	[45]
Cs_x_WO_3_@g‐C_3_N_4_/PVDF fiber membranes	2	95.4	0.92	2021	[46]
MXene/LSC hydrogels	1	92.3	2.73	2021	[47]
Co@C/NCNT‐based membrane	1	89.7	1.55	2022	[48]
Ta_2_O_5_/C‐HoMS	1	NA.	4.02	2022	[49]
MWCNTs‐ZrO_2_‐Ni@CQDs	1	89.5	1.95	2022	[50]
B@5/CNF hybrid paper	1	91.5	1.45	2022	This work
	3	81.2	3.88		

According to the World Health Organization (WHO), globally, at least 2 billion people drink bacteria‐contaminated water, and over fifty thousand people die from water‐borne infections annually. It is well acknowledged that millions of invisible pathogenic bacteria could thrive in the photothermal evaporation‐obtained water and cause deadly diseases, especially in developing countries, including dysentery, cholera, and typhoid fever.^[^ ^17^ ^]^ On the other hand, WHO notes that most pathogenic bacteria can be rapidly killed at temperatures above 149°F (65 °C), making thermal inactivation a practical approach for water sterilization. Our previous results indicate that the surface temperature of the B@5/CNF under three sun irradiation can reach above 67 °C, and therefore, it is expected that our borophene papers should also exhibit excellent photothermal antibacterial properties. In our experiment, we used the B@5/CNF paper for the inactivation of two typical bacteria as a demonstration, i.e., gram‐negative E. coli (ATCC MG2655) and gram‐positive B. subtilis (BS168).

**Figure 4:**
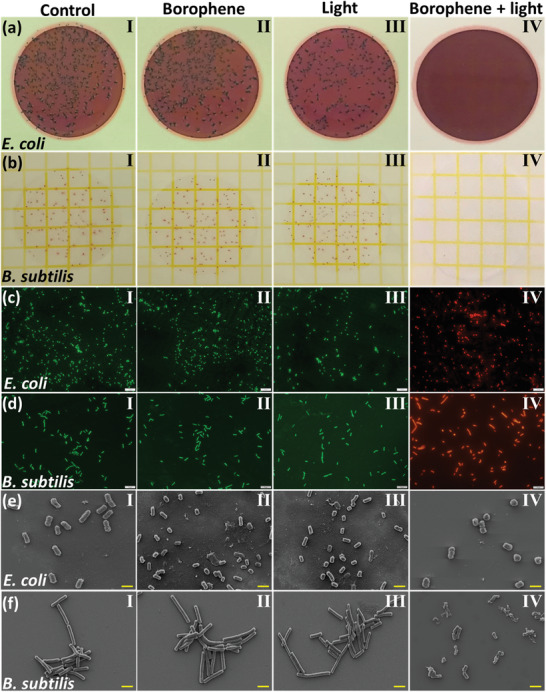
Antibacterial performance of four different groups. From left to right is Control group (pure bacterial under dark), Borophene group (add the B@5/CNF paper), Light group (bacterial under light illumination), and Borophene + light group, respectively. Photographs of a) *E. coli* and *B. subtili*s bacterial colonies with different treatments. The fluorescent images of c) *E. coli* and d) *B. subtili*s before and after different treatments. The scale bar is 2 µm. Their SEM images are shown in e)‐f). The scale bar is 1 µm.

In the beginning, we used the colony‐counting method to measure the antibacterial performance of borophene papers. As shown in **Figure** **4**a,b, no significant antibacterial effect can be observed in the light‐only and the no‐light borophene groups after 20 min compared to the control group, revealing that light illumination alone could not kill bacteria and the borophene paper does not have strong anti‐biofouling properties in the absence of light. In contrast, the borophene paper with irradiation group killed almost 100% of cells for both the tested bacterial strains after 20 min, owing to the rapid increase in temperature at the air‐water interface. These results were further confirmed by the live‐dead bacterial assay (Figure 4c,d), where the bacterial cells in the control, borophene, and light‐only groups showed green fluorescence of the dye (SYTO‐9), indicating nearly all of the bacteria were alive. On the contrary, bacterial cells in the B@5/CNF sample in the presence of light displayed remarkable red fluorescence from the dye (propidium iodide), suggesting almost all cells are dead. Note that bacteria can also form spores when exposed to higher temperatures and germinate again in favorable conditions. Thus, whether the thermal inactivation by B@5/CNF paper resulted in the complete killing of the bacteria or resulted in spore formation was tested by leaving the sample after exposure to the LB broth for 24 – 48 h. A complete lack of bacterial activity in the treated samples further confirms that the rapid heating during the photothermal treatment results in the complete killing of bacteria without any spore formation.

Morphology changes of the tested bacteria were characterized by SEM to investigate the antibacterial mechanisms of the borophene paper under irradiation conditions. As demonstrated in Figure 4e,f, all *E. coli* and *B. subtilis* cells in the control, borophene, and light‐only groups have intact and smooth surfaces. Nevertheless, their counterparts in the borophene paper with light illumination show severe cell membrane damage or are entirely broken. In addition, their size also significantly shrunk, possibly from the release of cellular components after cell wall damage, suggesting irreversible damage caused to the bacteria. Consequently, the antibacterial mechanism of borophene papers under light illumination should be attributed to the destruction of the cell wall by photothermal effects.

## 3 Conclusion

In summary, we propose a novel and facile strategy for fabricating flexible borophene papers by incorporating 2D borophene nanosheets into CNF paper, featuring excellent stability and mechanical properties. Thanks to the strong absorption ability, borophene papers demonstrate remarkable light‐to‐heat conversion efficiency of 91.5% and water evaporation rate of 1.45 kg m^−2^ h^−1^ under one sun irradiation. In particular, the borophene papers also illustrate good antibacterial performance, which can eliminate 100% of bacteria within 20 min under three sun irradiation, enabling a prospective future for highly efficient and long‐term solar energy‐driven photothermal applications.

## Conflict of Interest

The authors declare no conflict of interest.

## Supporting information

Supporting Information

## Data Availability

The data that support the findings of this study are available from the corresponding author upon reasonable request.
