# Supplementary material for: Borophene Embedded Cellulose Paper for Enhanced Photothermal Water Evaporation and Prompt Bacterial Killing
Source: Adv Sci (Weinh). 2023 Jan 25;10(7):2205809. doi: 10.1002/advs.202205809 (PMC9982542; doi:10.1002/advs.202205809)
Supplement: Supplementary file 1 — Supporting Information [file ADVS-10-2205809-s001.pdf]

ADVANCED SCIENCE
Open Access

Supporting Information

for *Adv. Sci.*, DOI 10.1002/advs.202205809

Borophene Embedded Cellulose Paper for Enhanced Photothermal Water Evaporation and Prompt Bacterial Killing

Xinwei Guan, Prashant Kumar, Zhixuan Li, Thi Kim Anh Tran, Sumit Chahal, Zhihao Lei, Chien-Yu Huang, Chun-Ho Lin, Jing-Kai Huang, Long Hu, Yuan-Chih Chang, Li Wang, Jolitta S. J. Britto, Logeshwaran Panneerselvan, Dewei Chu, Tom Wu, Ajay Karakoti, Jiabao Yi and Ajayan Vinu**

Supporting Information

Borophene Embedded Cellulose Paper for Enhanced Photothermal Water Evaporation and Prompt Bacterial Killing

Xinwei Guan, Prashant Kumar, Zhixuan Li, Thi Kim Anh Tran, Sumit Chahal, Zhihao Lei, Chien-Yu Huang, Chun-Ho Lin, Jing-Kai Huang, Long Hu, Yuan-Chih Chang, Li Wang, Jolitta S. J. Britto, Logeshwaran Panneerselvan, Dewei Chu, Tom Wu, Ajay Karakoti, Jiabao Yi, and Ajayan Vinu**

Experimental Section

***Materials Synthesis and Device Fabrication*:** Boron powder was purchased from Alfa Aesar (purity 99.5%). Freeze-dried CNF was purchased from Cellulose Lab. All other chemicals and solvents were purchased from Sigma-Aldrich and ACS reagent grade.

Freestanding borophene nanosheets were obtained by a sonochemical method, in which 100 mg of boron powder was dispersed in 40 ml of DMF solvent in a glass bottle. The bottle was then kept under a strong probe ultrasonic processor (Yuchengtech FS-1800N, 1800 W) and the dispersion was ultrasonicated for 12 hours. Afterward, the resulting fluid was centrifuged (Thermo Scientific, MEGAFUGE 8) at various speeds of 3000-5000 rpm for 5 minutes, and the supernatant was collected and characterized.

For the preparation of borophene nanofiber papers, CNF powders were dissolved in DMF to form 5 wt% CNF and totally dissolved by ultrasonication. Subsequently, different amounts of borophene powder were mixed with CNC solution and vigorously stirred for at least 30 minutes at room temperature. Afterward, the mixture was vacuum filtrated for 12 hours to form dried borophene papers.

***Measurements*:** All the samples were characterized in ambient conditions. The borophene nanosheets were characterized using TEM (JEM-F200, JEOL Ltd.) at 200 kV. XRD was studied on a Bruker D8 ADVANCE diffractometer with Cu K_α (λ = 1.5406 Å) radiation. The surface morphologies of borophene papers were carried out by FE-SEM (JEOL-7900F) with a voltage

setting of 5 kV. The surface morphologies of borophene nanosheets and papers were measured by AFM (Bruker Dimension ICON SPM) with a scan rate of 0.512 Hz. UV-Vis absorption spectra of borophene/CNF papers were obtained with a double-beam UV/Vis/NIR spectrophotometer (PerkinElmer, Lambda 1050).

Photothermal water evaporation was tested based on water mass loss using an electronic balance (Shimadzu, TX2202L). In short, a borophene paper with a diameter of 40 mm was floated on the water's surface (50 mL) in a 50 mL beaker. A Newport Xenon arc lamp (Model: 66902) with intensity of 1 kW m^{-2} and 3 kW m^{-2} at the top of the beaker was used as simulated sunlight. Afterward, the surface temperature was recorded by an IR camera (FLIR) for 60 minutes at intervals of 5 minutes. The room temperature was around 23 °C, and humidity was kept at ~ 50 %. Photothermal antibacterial tests were performed using colony counting, fluorescence staining, and morphological observation methods. The gram-negative *E. coli* (ATCC MG2655) and gram-positive *B. subtilis* (BS168) bacteria were grown separately in LB broth (LN, Luria/Miller) medium overnight at 37 °C. Subsequently, a subculture was prepared by adding 200 µL (*E. coli*) or 400 µL (*B. subtilis*) of the first culture to 20 mL LB broth and it was cultivated until reaching the desired bacterial concentration OD_{600} = 0.1 (which was confirmed by plate colony counting ~ 10^8 CFU/mL. Bacteria at a concentration of 1.0×10^6 CFU/mL was achieved by dilution with phosphate buffered saline (PBS, 10 mM, pH = 7.4), and 10 mL of the obtained bacterial solution was poured into a 25 mL glass beaker. Afterward, a borophene-embedded cellulose paper was placed on the surface of the bacteria solution, and then a three-sun illumination was applied to the solution for 10 min. An untreated bacteria solution was used as a blank control, whereas a borophene paper without irradiation and a pure bacteria culture solution with irradiation were used as borophene and lighting controls, respectively. After irradiation, 1 mL of the collected bacteria solution from each group was centrifuged, washed twice with PBS, and then resuspended in PBS for further use.

Colony-counting methods: To determine the number of bacteria colonies after the treatments, 100 μL of the above bacteria solution from each group was serially diluted 1000 times with PBS. Afterward, 1 mL diluted bacteria solution was spread on Petri films (3M Petrifilm E. coli/Coliform Count Plate and 3M™ Petrifilm™ Aerobic Count Plate for enumerating *E. coli* and *B. subtilis*, respectively) and incubated at 37 °C for 16 hours. Finally, bacterial colonies were enumerated by necked eyes.

Fluorescence-based viability assay: To further assess the thermal antibacterial effects of the borophene paper, the bacterial cell viability was assessed using LIVE/DEAD BacLight Bacterial Viability Kit (Cat# L7007). A 1.5 µL of dye solution mix containing SYTO-9 and propidium iodide (PI) was added into a 300 μL solution of the above bacteria from each group. The live and dead bacteria were stained green and red by SYTO-9 and PI, respectively. The stained bacteria were subsequently observed using a fluorescence microscope (Olympus IX 73, Japan).

Scanning Electron Microscope (SEM): Changes in bacteria morphology after treatments were observed using SEM (FE-SEM, JEOL-7900F). 5 µL of the four typical groups of bacterial suspensions were dropped on silicon wafers and fixed with 3 µL of 4% paraformaldehyde containing PBS for 30 min at room temperature. Then, the bacteria were dehydrated by sequential treatments with 30, 50, 70, 90, and 100% ethanol each for 5 min before coating with platinum for SEM observation.

Table S1. Water evaporation rates and solar steam efficiency of pure water, blank CNF paper, B@1/CNF paper, and B@5/CNF paper under one sun and three sun illumination.

	1 Sun		3 Sun	
	Efficiency (%)	Evaporation rate (kg $m^{-2}h^{-1}$)	Efficiency (%)	Evaporation rate (kg $m^{-2}h^{-1}$)
Pure water	23.9	0.38	19.9	0.95
CNF blank	24.8	0.39	21.3	1.02
B@1/CNF	58.4	0.93	46.2	2.21
B@5/CNF	91.5	1.45	81.2	3.88

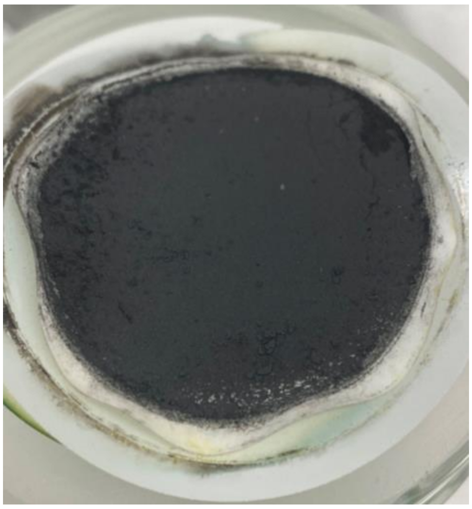

Figure S1. Digital photo of borophene depositing on the surface of cellulose paper. External forces like scratching and folding could easily wipe out the borophene powder.

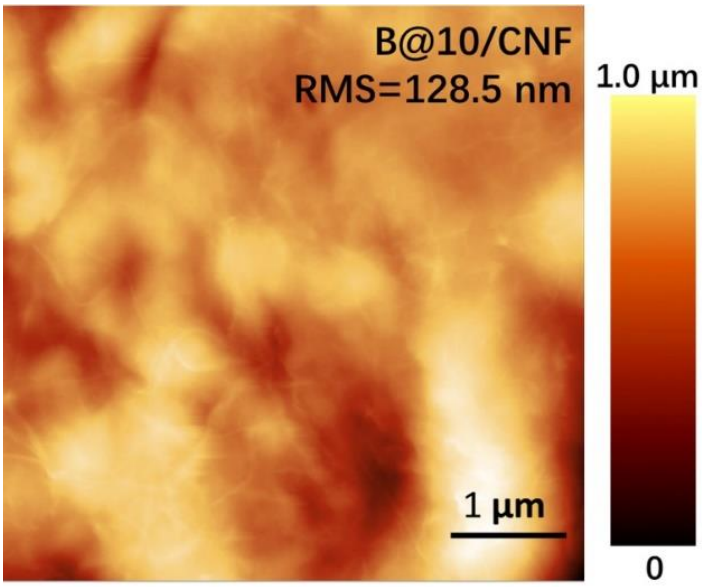

Figure S2. AFM image of the B@10/CNF paper.

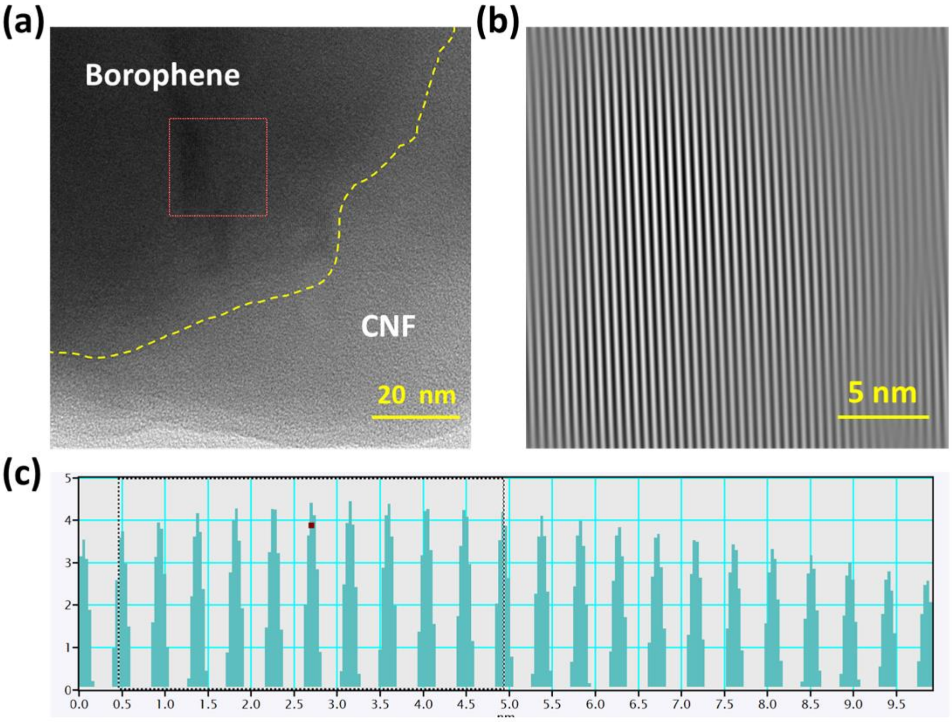

Figure S3. a) TEM image of the B@5/CNF hybrid paper and b) the zoomed-in HRTEM image. c) The average interatomic distances at two locations were 0.50 nm, which is consistent with borophene nanosheet results.

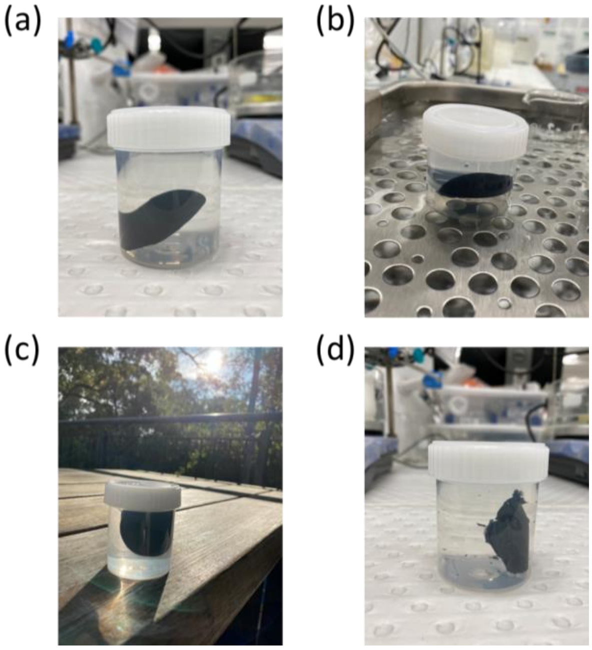

Figure S4. a) Digital photos of the B@5/CNF paper just immersed in water, b) After ultrasonicated for 1 hour, and c) immersed in water for one month outdoors. No visible difference or broken paper pieces can be observed. d) B@10/CNF paper immersed in water. Gently shaking the solution caused damage to the paper, and we can observe black paper scraps in the water solution.

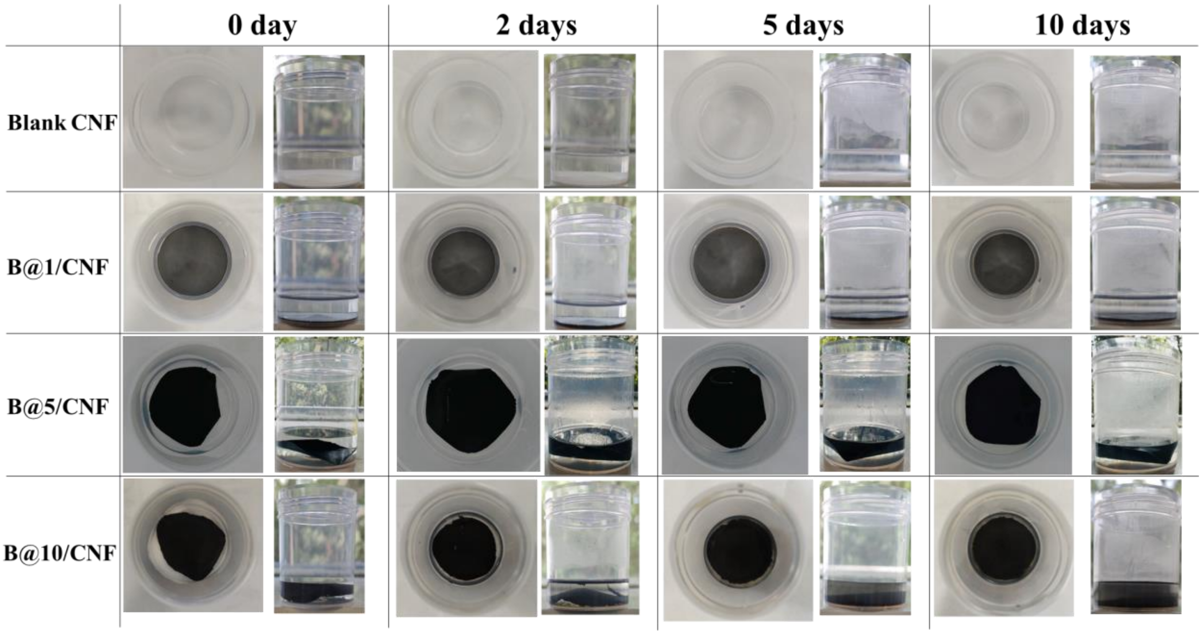

Figure S5. Digital photos of the pure CNF, B@1/CNF, B@5/CNF, and B@10/CNF papers immersed in water for ten days.

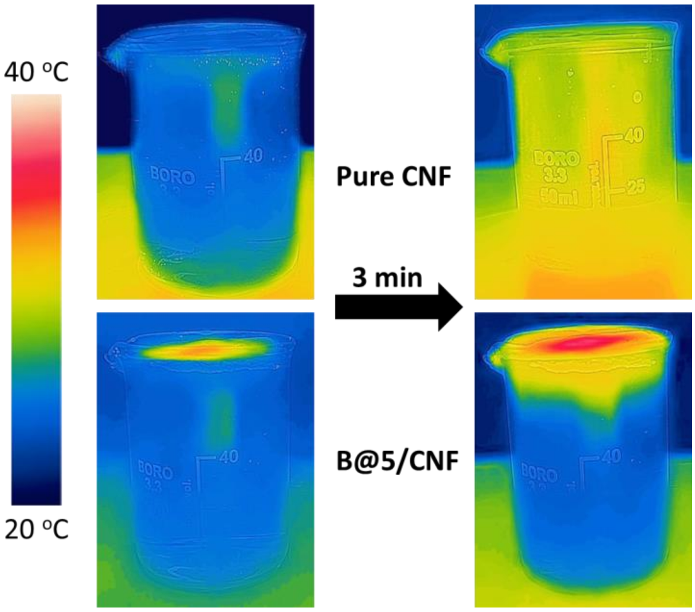

Figure S6. Side-view IR thermal images of pure CNF paper and B@5/CNF paper under one sun irradiation for 3 minutes.

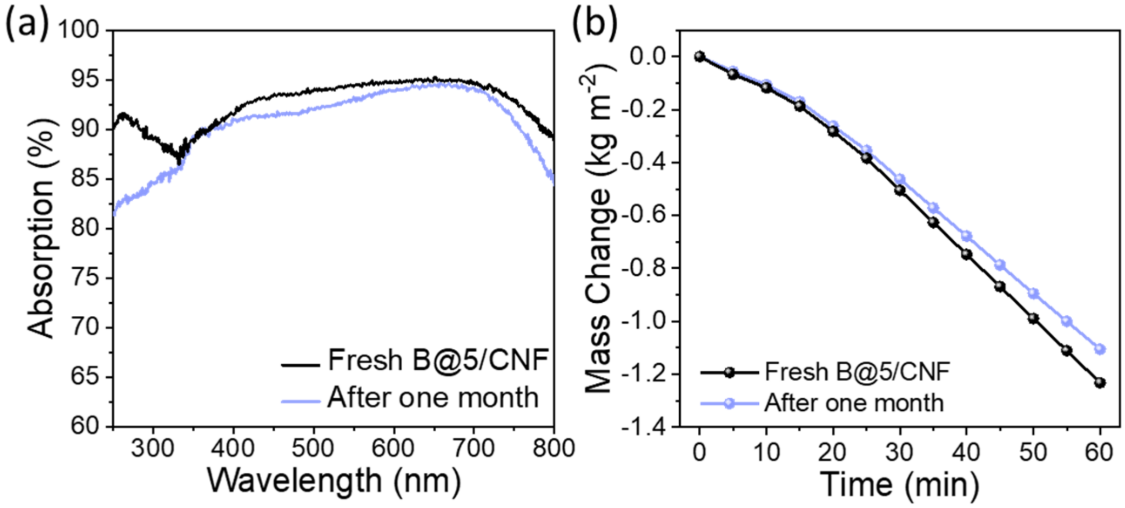

Figure S7. a) UV-Vis absorption spectrum of fresh B@5/CNF and after one-month aging. b) water mass changes of fresh B@5/CNF and paper after one month under one sun irradiation.

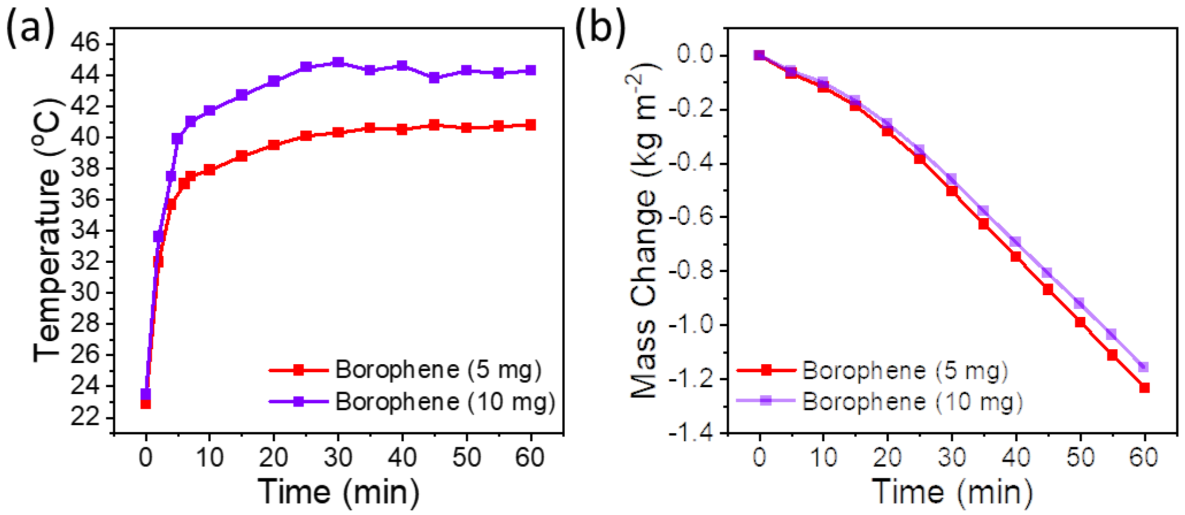

Figure S8. a) Temperature change of B@5/CNF and B@10/CNF, and b) water mass changes of these two papers under one sun irradiation. Although the B@10/CNF results in a higher equilibrium temperature, the water mass change is less than the B@5/CNF paper.

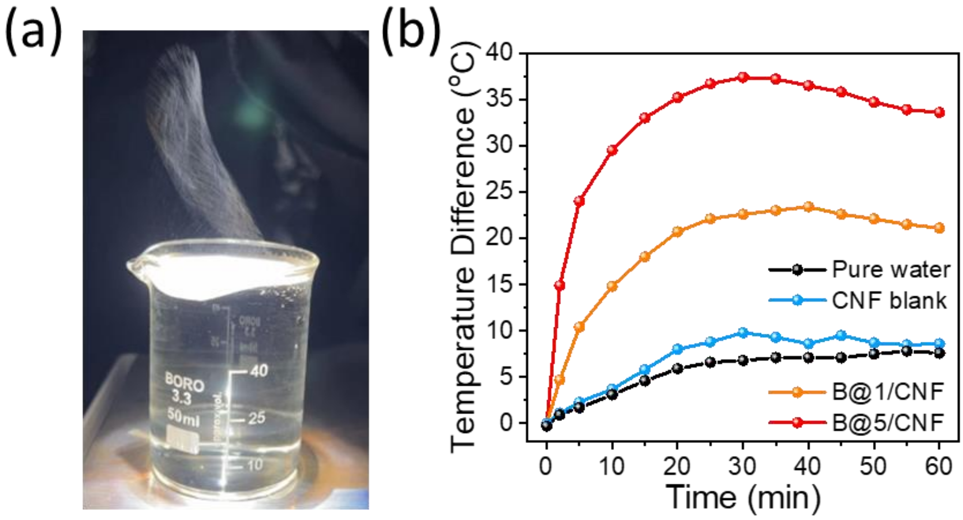

Figure S9. a) Digital image of steam generation under one sun illumination. b) The temperature difference between the top paper surface and the beaker's bottom of the surface of pure water, blank CNF paper, B@1/CNF paper, and B@5/CNF paper under three sun irradiation.
